# Supplementary material for: Ultrasensitive detection of clinical pathogens through a target-amplification-free collateral-cleavage-enhancing CRISPR-CasΦ tool
Source: Nat Commun. 2025 Apr 26;16:3929. doi: 10.1038/s41467-025-59219-x (PMC12032082; doi:10.1038/s41467-025-59219-x)
Supplement: Supplementary file 1 — Supplementary Information [file 41467_2025_59219_MOESM1_ESM.pdf]

Supplementary Information

Ultrasensitive Detection of Clinical Pathogens Through a Target-Amplification-free Collateral-cleavage-enhancing CRISPR-CasΦ Tool

Huiyou Chen[1,2,3,†], Fengge Song[1,2,3,†], Buhua Wang[1,3], Hui Huang[4], Yanchi Luo[1,3], Xiaosheng Han[4], Hewen He[5], Shaolu Lin[5], Liudang Wan[5], Zhengliang Huang[5], Zhaoyong Fu[5], Rodrigo Ledesma-Amaro[6], Dapeng Yin[7], Haimei Mao[8], Linwen He[9], Tao Yang[10], Zijing Chen[10], Yubin Ma[10], Evelyn Y. Xue[10], Yi Wan[1,3,11,#], Chuanbin Mao[10,#]

[1]State key laboratory of digital medical engineering, Hainan University, Haikou 570228, China.

[2]School of Life and Health Sciences, Hainan University, Haikou 570228, China.

[3]State Key Laboratory of Marine Resource Utilization in South China Sea, Hainan University, Haikou 570228, China.

[4]Microbial Medical Laboratory, People's Hospital of Haikou, Haikou, 570208, China.

[5]Hainan Viewkr Biotechnology Co., Ltd, Haikou, 570228, China

[6]Imperial College Centre for Synthetic Biology, Department of Bioengineering, Imperial College London, London SW7 2AZ, United Kingdom.

[7]Hainan Center for Disease Control and Prevention, Haikou 570228, China.

[8]Products Quality Supervision and Testing Institute of Hainan Province, Haikou 570003, China.

[9]School of Marine Biology and Fisheries, Hainan University, Haikou 570228, China.

[10]Department of Biomedical Engineering, The Chinese University of Hong Kong, Sha Tin, Hong Kong Special Administrative Region.

[11]School of Biomedical Engineering, Hainan University, Haikou 570228, China.

†These authors contributed equally to this work

[#]Corresponding Authors

E-mail address: 993602@hainanu.edu.cn (Y. Wan); cmao@cuhk.edu.hk (C. Mao)

Contents

Ultrasensitive Detection of Clinical Pathogens Through a Target Amplification-free Collateral-cleavage-enhancing CRISPR-CasΦ Tool 1

Supplementary Figure 1 | Purification and activity validation of CasΦ. 3

Supplementary Figure 2 | Optimization of the basic fluorescence detection system of CasΦ. 4

Supplementary Figure 3 | Screening and optimization of CasΦ gRNA sequences and lengths. 5

Supplementary Figure 4 | NUPACK-predicted secondary structures and Gibbs free energies for gRNA binding with ssDNA activators of different lengths. 6

Supplementary Figure 5 | Screening of 5' and 3' deletion and blocking of CasΦ activators. 7

Supplementary Figure 6 | Screening and verification of CasΦ blocking by different types of dsDNA activators. 8

Supplementary Figure 7 | Detection of *trans*-cleavage activation efficiencies by 5' and 3' toehold dsDNA activators. 8

Supplementary Figure 8 | Design and optimization of secondary structures for the TCC amplifier. 9

Supplementary Figure 9 | NUPACK-predicted 3' toehold DNA amplifier with a loop size of 6 nt (a), 8 nt (b), 10 nt (c), 12 nt (d). 10

Supplementary Figure 10 | Enzymatic reaction kinetics of CasΦ *trans*-cleavage of TCC amplifier and reporter. 11

Supplementary Figure 11 | Enzymatic reaction kinetics of CasΦ activated by dsDNA or ssDNA. 11

Supplementary Figure 12 | Optimization of TCC reaction conditions. 12

Supplementary Figure 13 | Ultra-sensitivity and high specificity of one-step TCC 13

Supplementary Figure 14 | Characterization of pathogen thermolysis detection by TCC and qPCR. 14

Supplementary Figure 15 | Detection of different pathogens using TCC 15

Supplementary Figure 16| qPCR and TCC quantification detection of simulated *E. coli* samples. 15

Supplementary Figure 17 | Detection of *E. coli* in serum by TCC and qPCR 16

Supplementary Figure 18 | Fluorescence kinetics detection of different bacterial species from BSI patients. 16

Supplementary Figure 19 | Principle and feasibility detection of the TCC Device 17

Supplementary Figure 20 | Point-of-care (POC) diagnosis of *E. coli* in serum by our own portable TCC Device 18

References 18

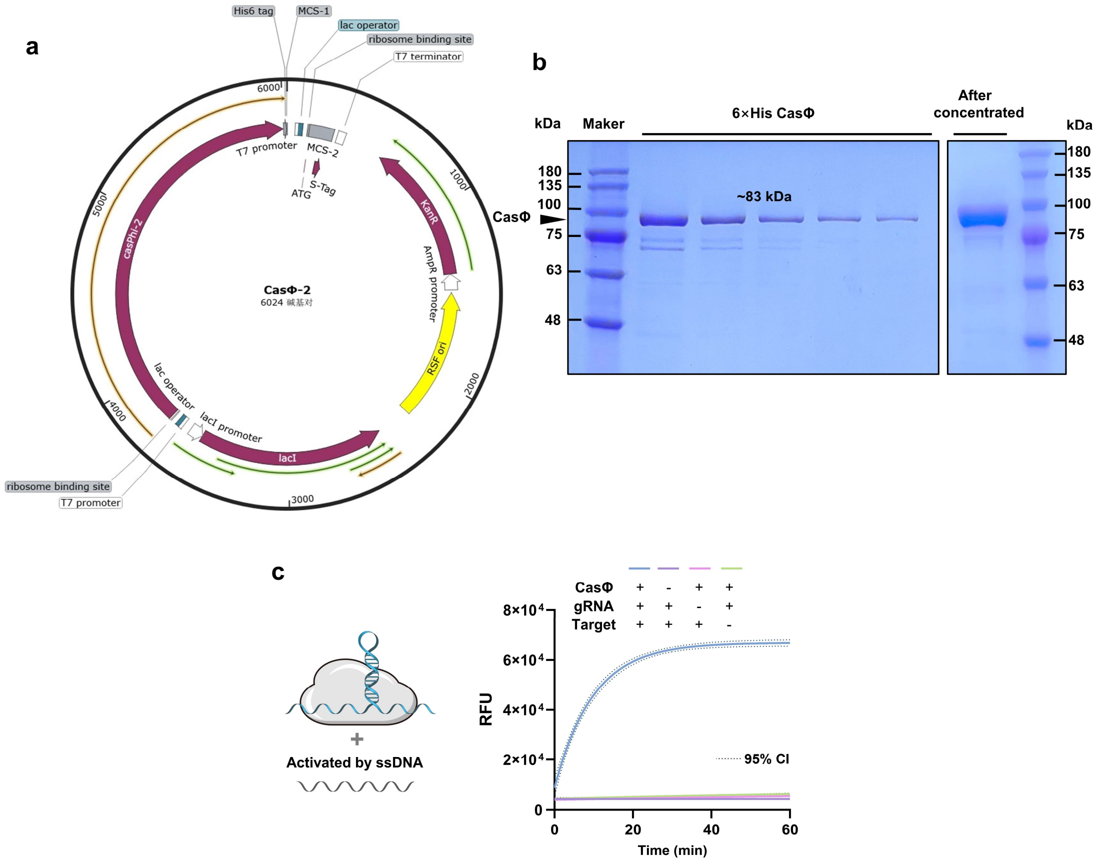

Supplementary Figure 1 | Purification and activity validation of CasΦ.

a, Plasmid map of CasΦ-2 (Addgene Plasmid #158795). **b**, Purified CasΦ was characterized by SDS-PAGE. The 6×His CasΦ is around 83 kDa. **c**, Activity validation of CasΦ using a ssDNA activator. Curve fitting was performed by averaging three independent biological experiments (n=3), and the dotted lines indicate 95% confidence intervals.

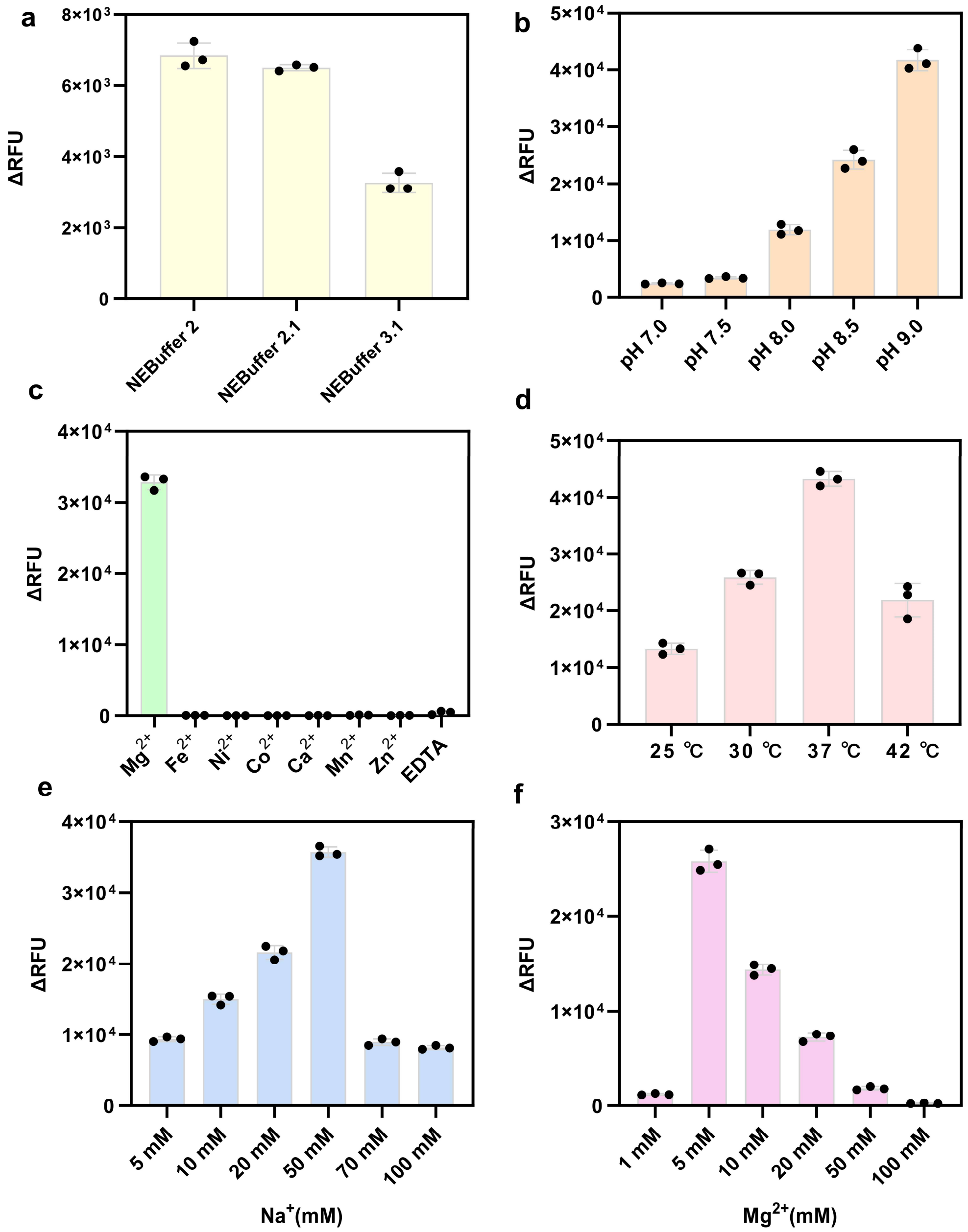

Supplementary Figure 2 | Optimization of the basic fluorescence detection system of CasΦ.

a, Activity measurement of CasΦ in NEBuffer. Showing CasΦ has the same activity in NEBuffer 2.0 and 2.1. **b**, Activity measurement of CasΦ under different pH. **c**, Divalent cation optimization for CasΦ, showing CasΦ only has *trans*-cleavage activity under Mg^{2+}. **d**, Reaction temperature optimization for CasΦ. **e**, Activity measurement of CasΦ under different NaCl concentrations, with 50 mM NaCl having the best activity. **f**, Optimization of CasΦ activity under different MgCl concentrations. All assays were performed with 3 independent technical repeats (n=3), error bars represent mean ± SD.

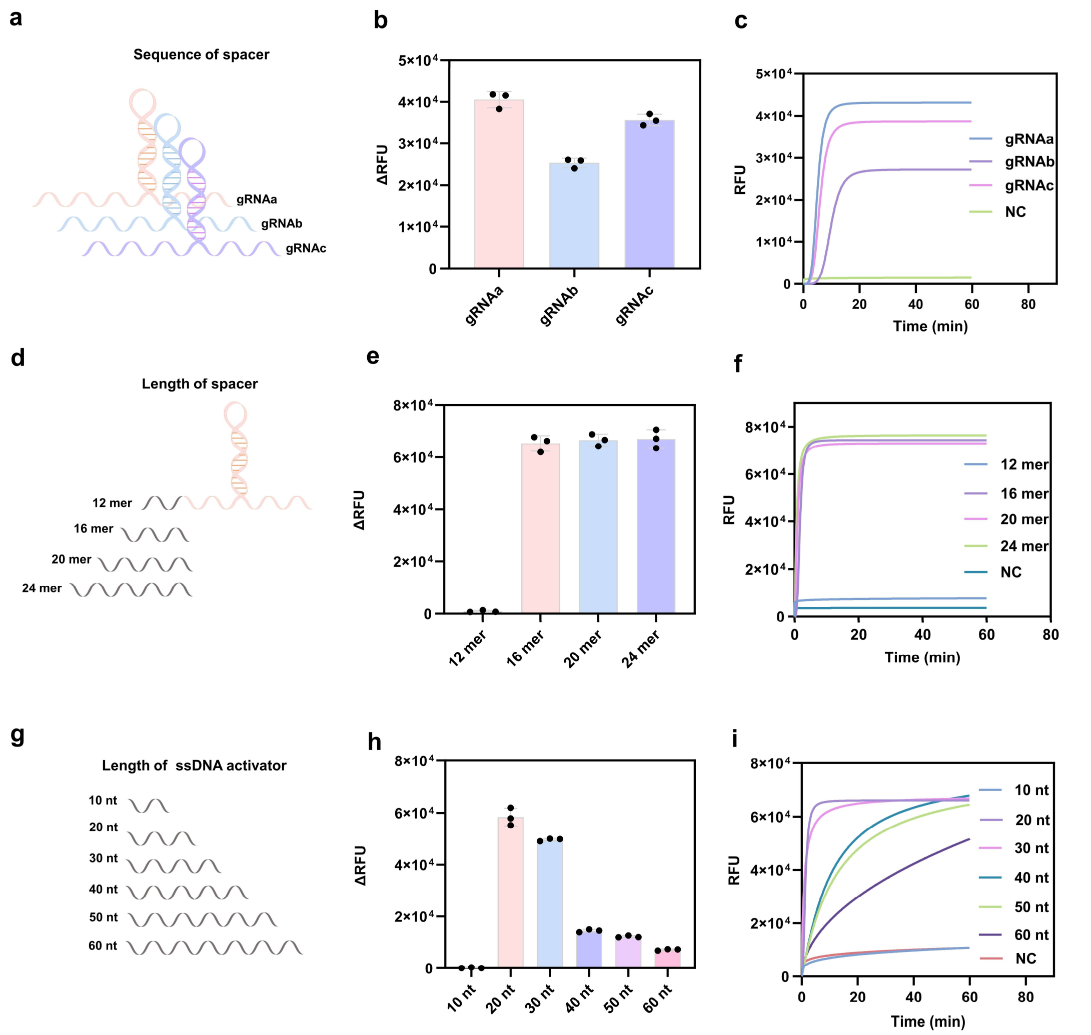

Supplementary Figure 3 | Screening and optimization of CasΦ gRNA sequences and lengths.

a, Schematics of 3 gRNAs with different sequences, gRNAa, gRNAb and gRNAc. **b** and **c**, Measurement of *trans*-cleavage efficiencies of CasΦ activated by gRNAs with different sequences, showing gRNAa has the best efficiency. **d**, Optimization schematics of gRNA spacer region length. **e** and **f**, Measurement of *trans*-cleavage efficiencies of CasΦ activated by gRNAs with different spacer lengths. 16 mer, 20 mer and 24 mer all have similar activation efficiencies. **g**, Schematic diagram of ssDNA activator length optimization screening. **h** and **i**, Measurement of *trans*-cleavage efficiencies of gRNAa-activated CasΦ by ssDNA activators with different lengths, showing 20nt ssDNA activator has the best efficiency. NC, DEPC H_2O as negative control. All assays were performed with 3 independent technical repeats (n=3), error bars represent mean ± SD. Kinetic curves were fitted using mean values.

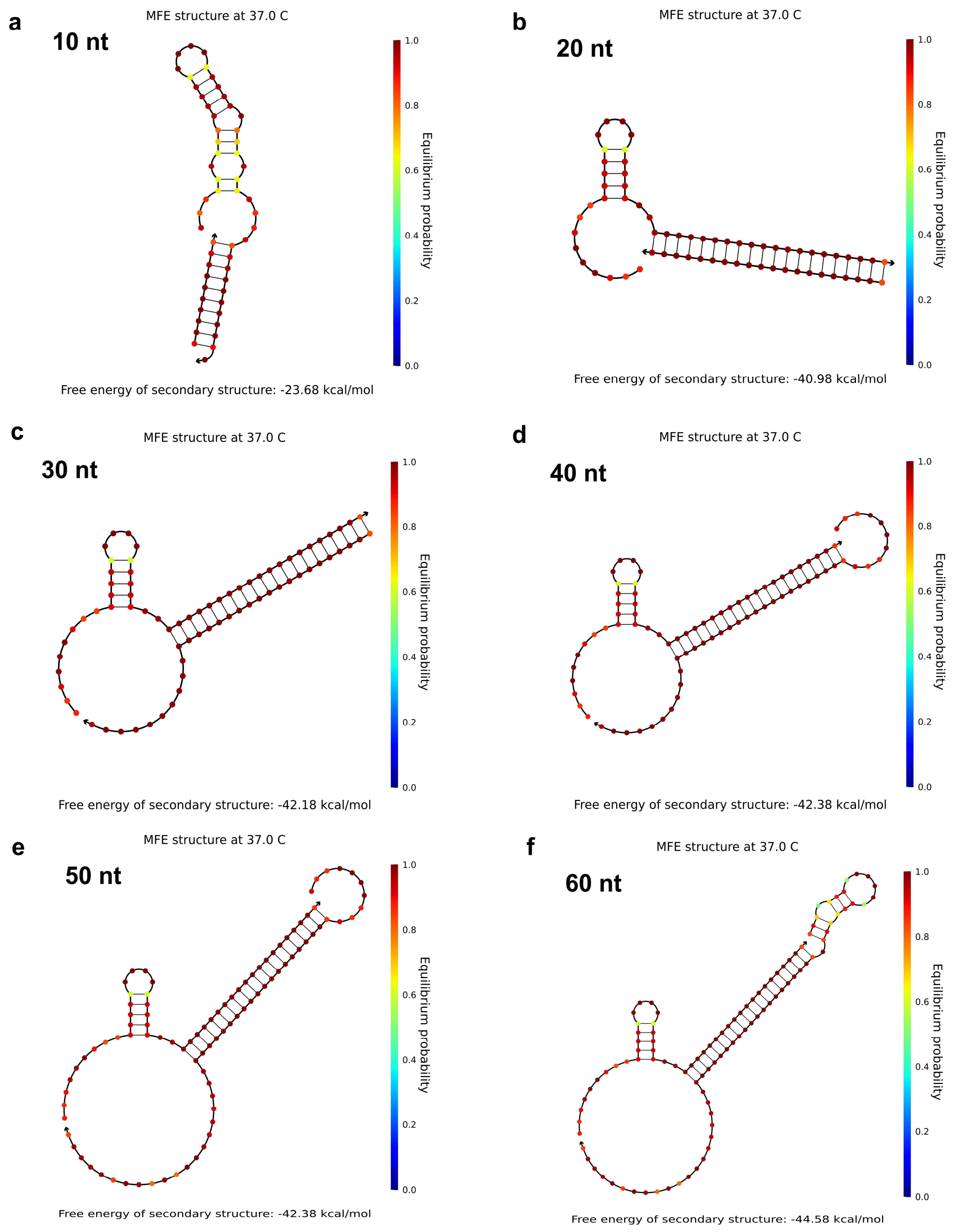

Supplementary Figure 4 | NUPACK-predicted secondary structures and Gibbs free energies for gRNA binding with ssDNA activators of different lengths.

a-f, Predictions for 10 nt, 20 nt, 30 nt, 40 nt, 50 nt, 60 nt ssDNA activators binding with gRNA, respectively. Parameters were set as 37 °C, Na^{+} (50 mM), Mg^{2+} (10 mM), gRNA (100 nM), ssDNA activators (100 nM).

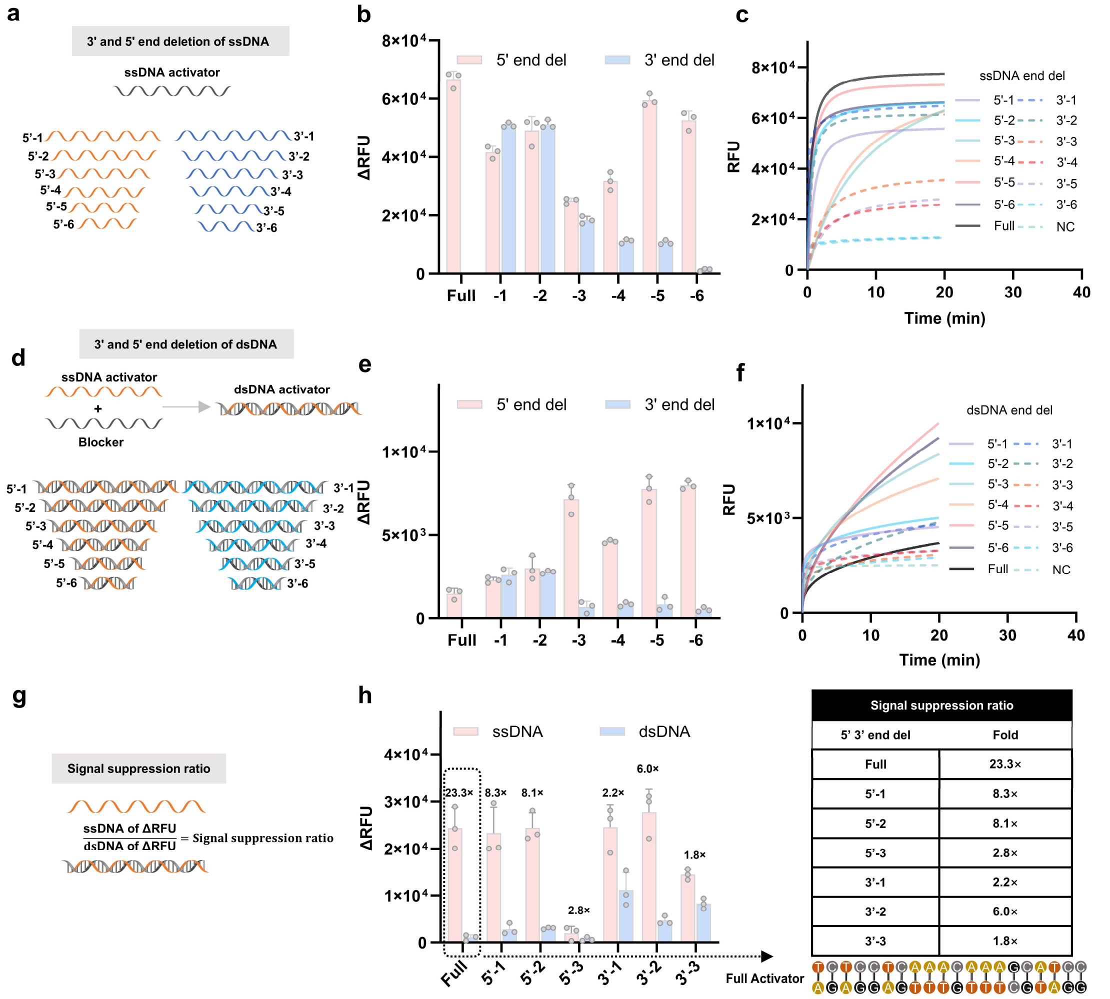

Supplementary Figure 5 | Screening of 5’ and 3’ deletion and blocking of CasΦ activators.

a, Schematics of 5’ and 3’ deletion of ssDNA activator. **b**, Fluorescence detection of 25 nM ssDNA activator with 5’ and 3’ deletion. Full, full-length ssDNA activator. **c**, Fluorescence kinetics of ssDNA activator deletion. NC, H_2O as negative control. **d**, Schematics of 5’ and 3’ deletion and blocking of dsDNA activator. Blocking was done using a Blocker ssDNA fully complementary to ssDNA activator. **e**, Fluorescence detection of 2.5 nM dsDNA activator with 5’ and 3’ deletion. **f**, Fluorescence kinetics of dsDNA activator deletion and blocking. **g**, Formula for signal suppression fold. Calculated by dividing fluorescence signal ΔRFU of ssDNA activator by ΔRFU of dsDNA activator. **h**, Suppression folds at 25 nM activator concentration. Showing full target sequence has the highest signal suppression fold. Bar charts in b, e and h used t=5 min endpoint fluorescence for data processing and plotting. Curve fitting in e and f was done by averaging three independent technical repeats. Error bars represent mean ± SD.

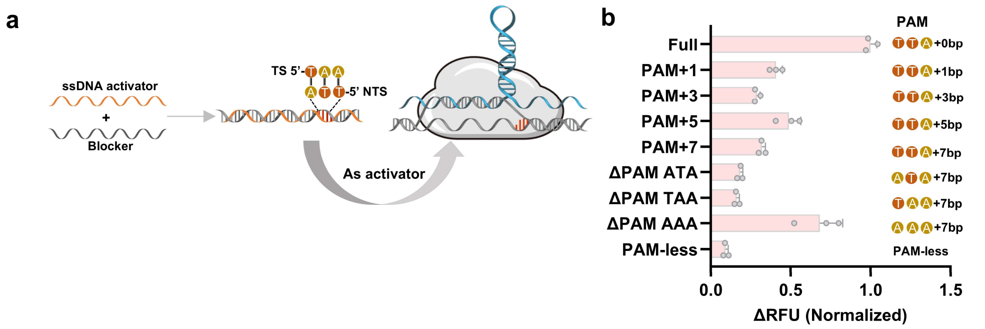

Supplementary Figure 6 | Screening and verification of CasΦ blocking by different types of dsDNA activators.

a and **b**, Measurement of blocking effects on CasΦ by different types of dsDNA activators. Full indicates full-length dsDNA sequence containing PAM. PAM less, 20 bp dsDNA activator without PAM site. PAM+1, dsDNA with 1bp extension from 3' end containing PAM. PAM+3, PAM+5, PAM+7 and so on. ΔPAM, PAM TTA of CasΦ mutated to other bases. All assays were performed in 3 independent biological triplicates (n=3), error bars represent mean ± SD.

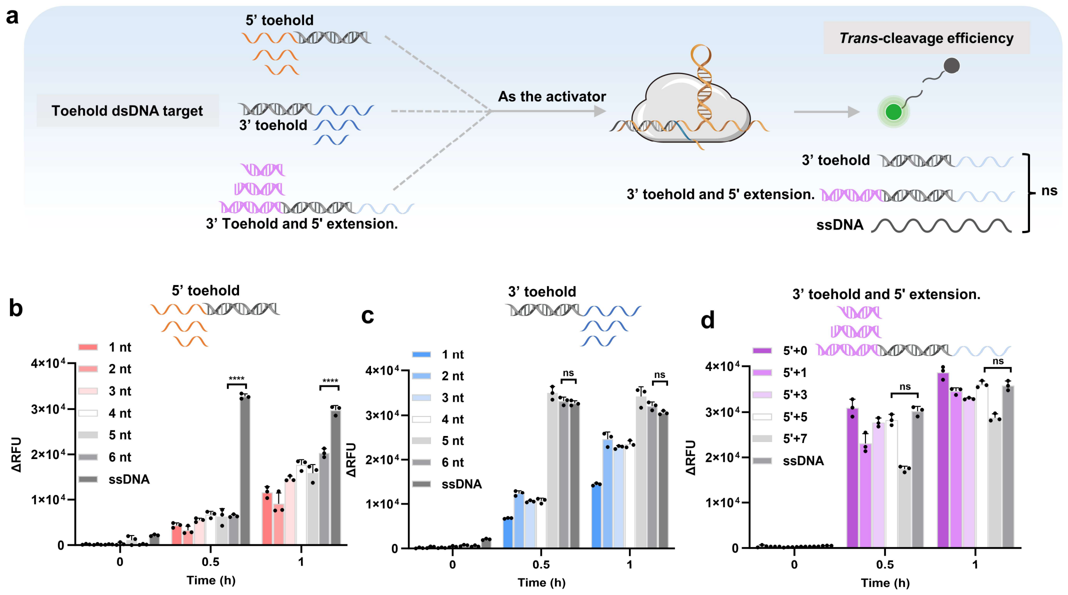

Supplementary Figure 7 | Detection of *trans*-cleavage activation efficiencies by 5' and 3' toehold dsDNA activators.

a, Schematics showing activation of CasΦ by different types of toehold dsDNA. Orange indicates the 5' toehold sequences of different lengths, blue indicates the 3' toehold sequences of different lengths, purple indicates the 5' extension sequences of different lengths. **b**, Activation efficiency measurement of 5' toehold dsDNA. ssDNA indicates full-length ssDNA activator. **c**, Activation efficiency measurement of 3' toehold dsDNA. **d**, Activation efficiency measurement of dsDNA with 3' toehold and 5' extension. One-way ANOVA and Turkey's multiple comparisons test were performed on 3 independent biological triplicates (n=3) for each group. ns = not significant > 0.05. Asterisks indicate significant differences (**p* ≤ 0.05, ***p* ≤ 0.01, ****p* ≤ 0.001, *****p* ≤ 0.0001). Error bars represent mean ± SD.

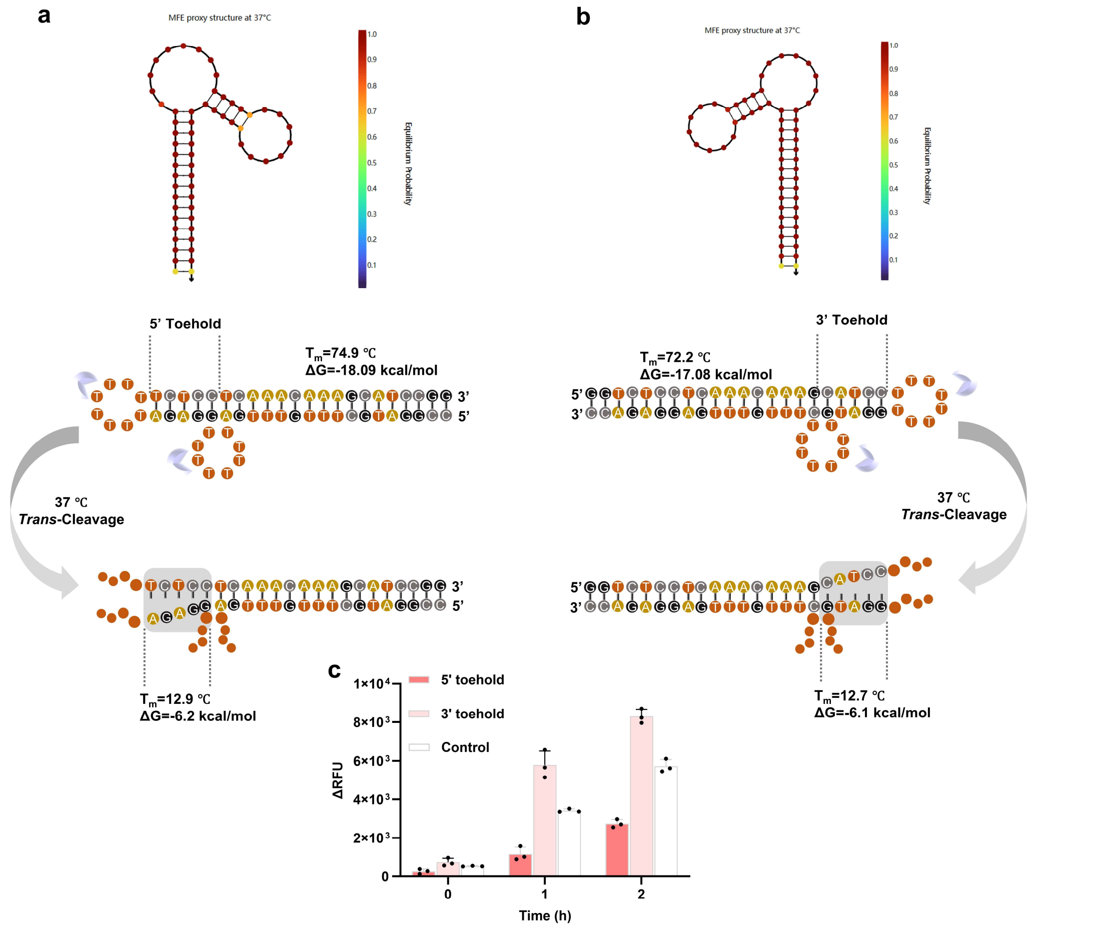

Supplementary Figure 8 | Design and optimization of secondary structures for the TCC amplifier. **a** and **b**, NUPACK-predicted structures and DINAMelt-calculated TM values and Gibbs free energies for 5' (a) and 3' (b) toehold amplifiers. NUPACK and DINAMelt parameters were uniformly set as 37 °C, Na^{+} (50 mM), Mg^{2+} (10 mM), amplifiers (100 nM). **c**, Comparison of cascading reactions of 5' and 3' toehold amplifiers. Control, 10 pM dsDNA activator. Assays were performed in 3 independent technical repeats (n=3), error bars represent mean ± SD.

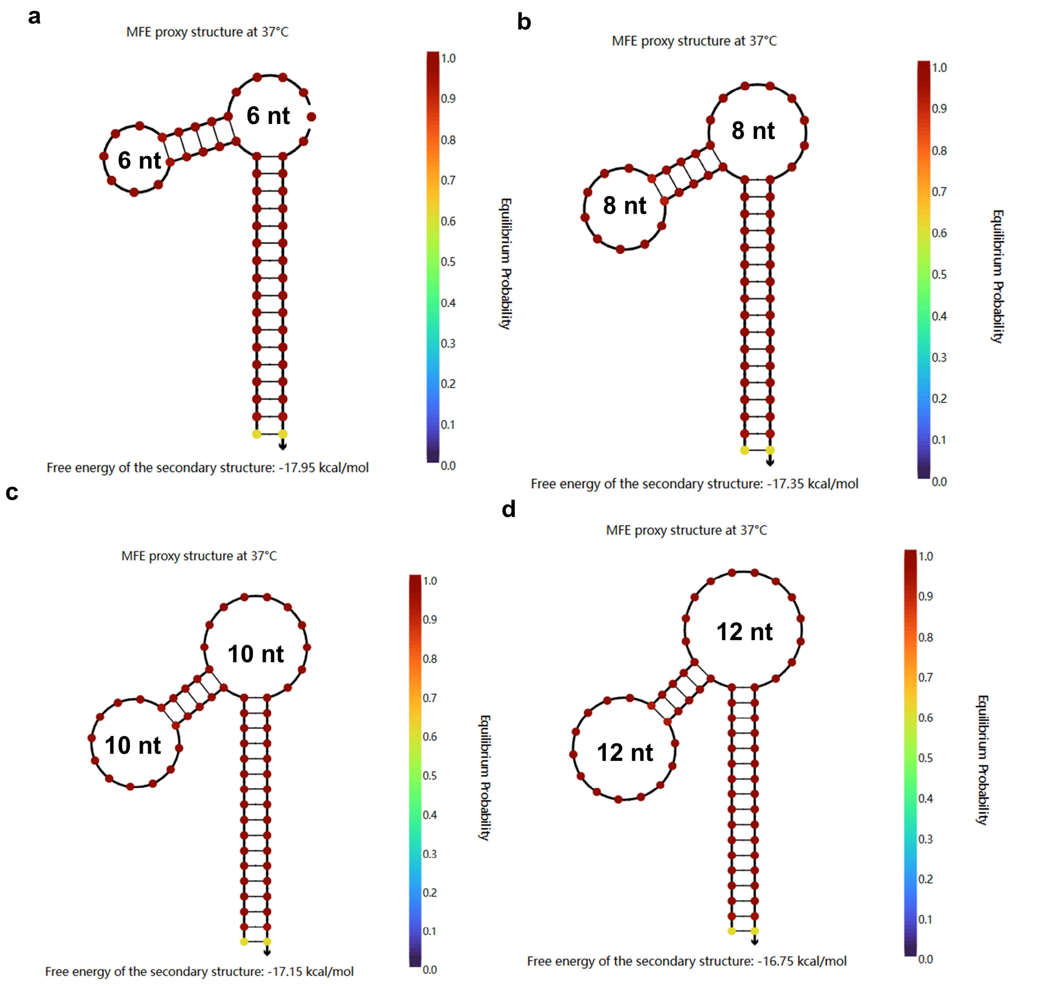

Supplementary Figure 9 | NUPACK-predicted 3' toehold DNA amplifier with a loop size of 6 nt (a), 8 nt (b), 10 nt (c), 12 nt (d).

The NUPACK parameters were uniformly set as 37 °C, Na^{+} (50 mM), Mg^{2+} (10 mM), amplifiers (100 nM).

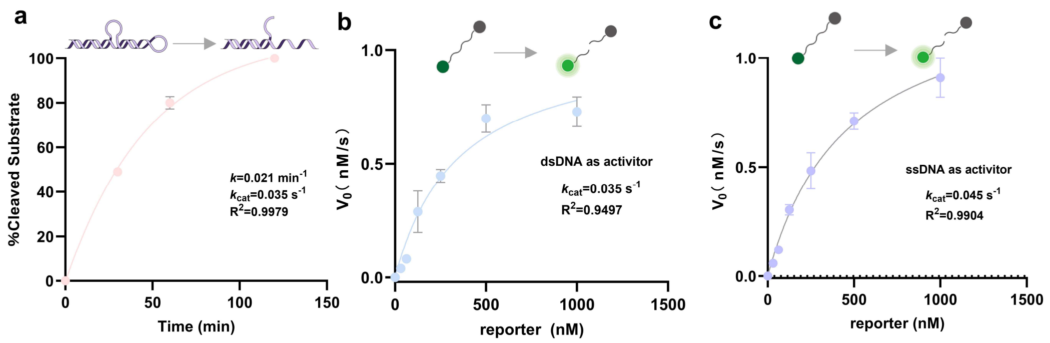

Supplementary Figure 10 | Enzymatic reaction kinetics of CasΦ *trans*-cleavage of TCC amplifier and reporter.

a, Enzymatic time-percentage cleavage kinetics of TCC amplifier. Cleavage percentage was calculated by grayscale values of native-PAGE bands in Fig. 2d. One phase decay equation was used for curve fitting to obtain the rate constant k, which was used to calculate k_{cat} of CasΦ *trans*-cleaving TCC amplifier[1]. Reactions were performed in 2 independent biological duplicates (n=2), error bars represent mean ± SD. **b**, Enzymatic reaction kinetics of CasΦ *trans*-cleaving reporter activated by dsDNA. Supplementary Figure 11a-c provide fluorescence kinetics and equations for calculating k_{cat}. **c**, Enzymatic reaction kinetics of CasΦ *trans*-cleaving the linker between fluorescent quenching and reporter activated by ssDNA. Supplementary Figure 11d-f provide fluorescence kinetics and equations for calculating k_{cat}. Calculation methods refer to Ramachandran, A. et al[2]. Reactions in b and c were performed in 3 independent biological triplicates (n=3), error bars represent mean ± SD.

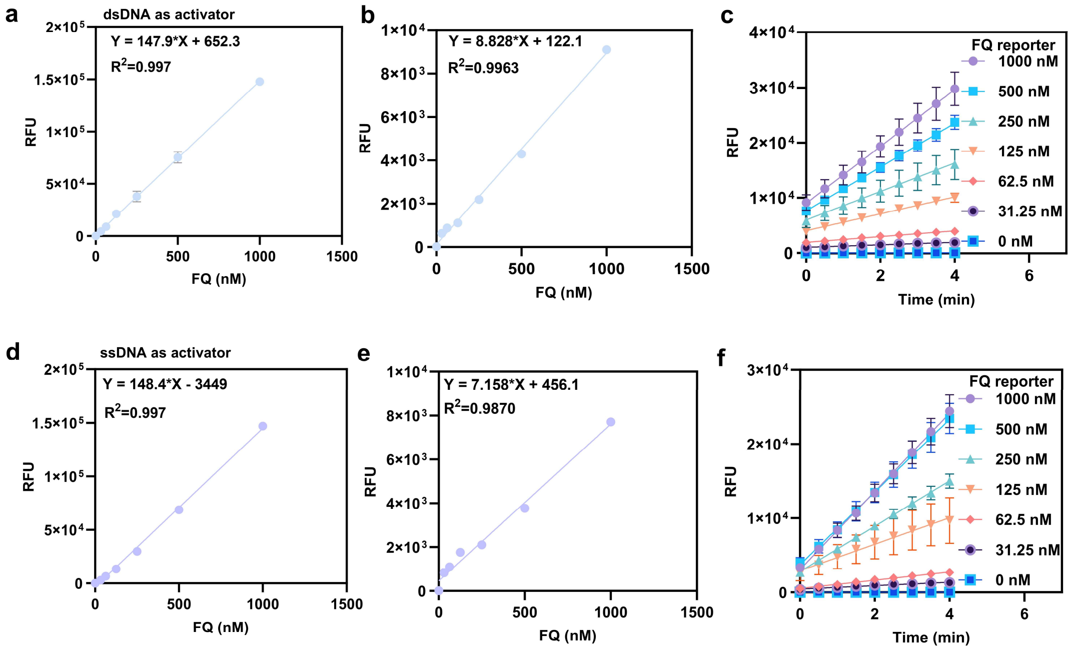

Supplementary Figure 11 | Enzymatic reaction kinetics of CasΦ activated by dsDNA or ssDNA.

a, Linear correlation between fluorescence and concentrations of the Reporter substrate cleaved by dsDNA-activated CasΦ. **b**, Linear correlation for the DEPC H_2O background corresponding to a. **c**, First-order reaction of Reporter cleavage catalyzed by dsDNA-activated CasΦ. **d**, Linear

correlation between fluorescence and concentrations of the Reporter cleaved by ssDNA-activated CasΦ. **e**, Linear correlation for the DEPC H_2O background corresponding to d. **f**, First-order reaction of Reporter cleavage catalyzed by ssDNA-activated CasΦ. Catalytic site concentration (Et= 10 nM). All assays were performed in three independent technical replicates (n=3), with error bars representing mean ± SD.

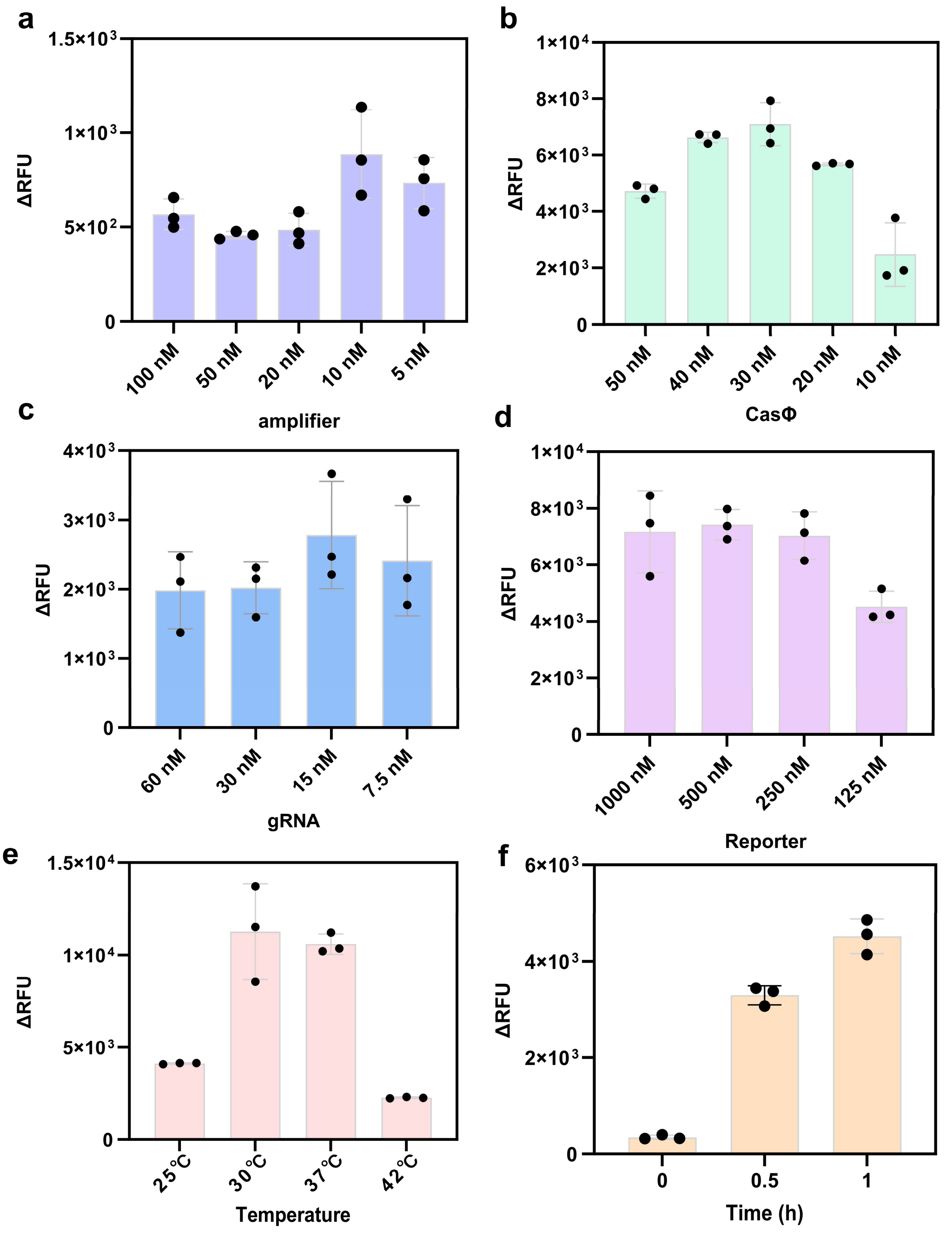

Supplementary Figure 12 | Optimization of TCC reaction conditions.

a, Optimization of TCC amplifier concentration. **b**, Optimization of CasΦ protein concentration. **c**, Optimization of gRNA concentration. **d**, Optimization of reporter concentration. **e**, Optimization of TCC reaction temperature. **f**, Measurement of TCC reaction time. The finalized reaction conditions are 10 nM TCC amplifier, 30 nM CasΦ protein, 15 nM gRNA, 250 nM reporter, 37 °C reaction temperature. All assays were performed in 3 independent biological triplicates (n=3),

error bars represent mean ± SD.

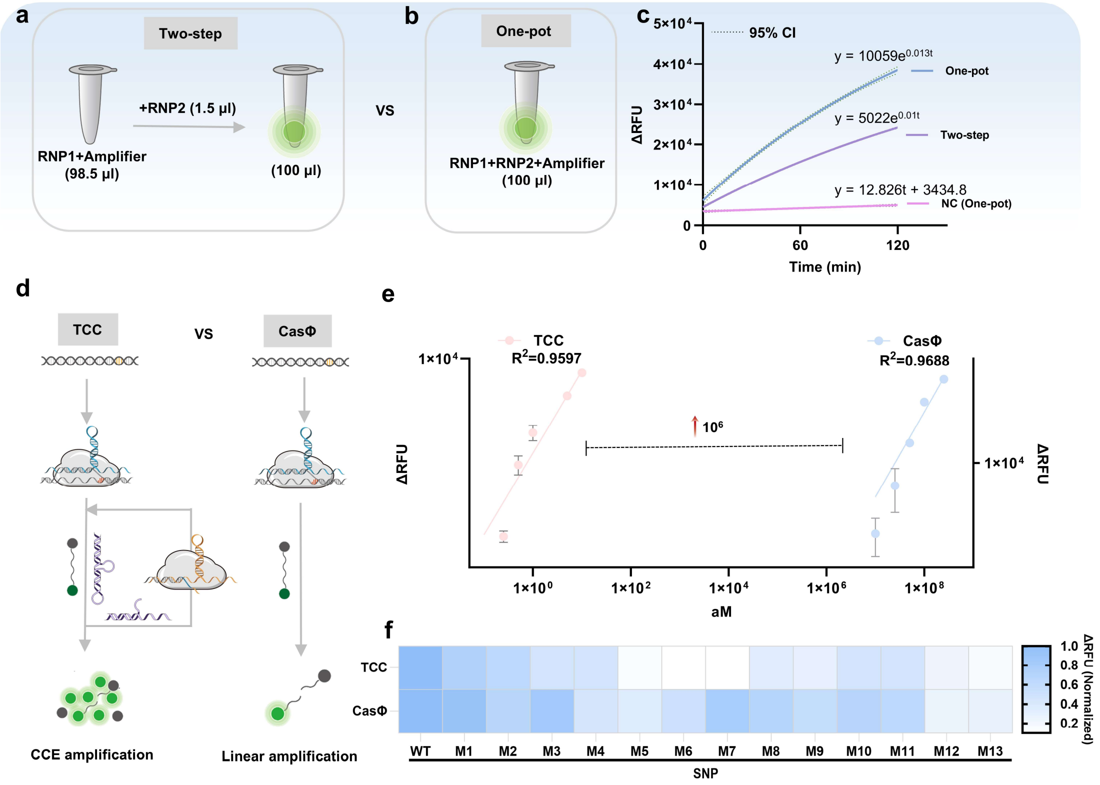

Supplementary Figure 13 | Ultra-sensitivity and high specificity of one-step TCC.

a-**c**, Comparison between two-step and one-step TCC. **a**, Two-step involves pre-cleavage of TCC amplifier by RNP1 for 30 min followed by adding RNP2 and the reporter in one-pot reaction. **b**, One-step directly mixes all reaction components in one pot. **c**, Fluorescence kinetics were fitted by averaging three technical repeats (n=3), fitting equation has y as RFU at specific time (t) and e as natural constant. NC, Replace the activator with DEPC H_2O (one-pot). **d**, Signal amplification pattern comparison between TCC and CasΦ. **e**, LOD linearity comparison between TCC and CasΦ in detecting dsDNA activator. Showing at least 6 orders of magnitude improvement in detection limit by TCC. Error bars represent mean ± SD. **f**, SNP discrimination comparison between TCC and CasΦ for dsDNA activator, showing higher specificity of TCC over CasΦ. WT, no mutation in dsDNA; M1-M12, SNP at each site. All assays were performed in three independent biological triplicates (n=3).

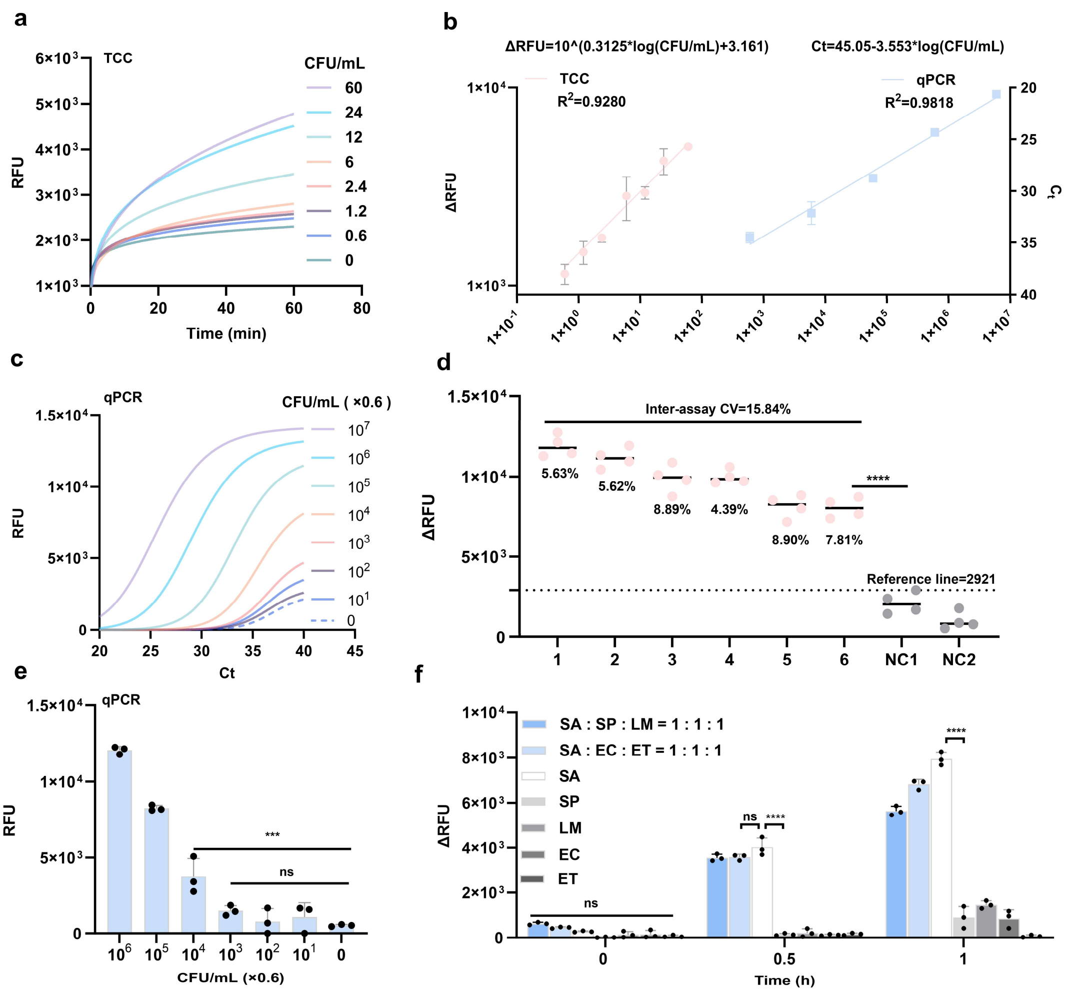

Supplementary Figure 14 | Characterization of pathogen thermolysis detection by TCC and qPCR.

a, Fluorescence kinetics of *S. aureus* detection by TCC. **b**, LOD linearity comparison between TCCand qPCR detecting *S. aureus*. Linear equations are ΔRFU=10^(0.3125*log(CFU/mL)+3.161) for TCC and Ct=45.05-3.553*log(CFU/mL) for qPCR. **c**, Fluorescence kinetics of *S. aureus* detection by qPCR. **d**, Repeatability and reproducibility of TCC detecting *S. aureus*, showing less than 10% intra-assay CV and less than 20% inter-assay CV. 24 CFU/mL *S. aureus* was tested. Each point is an average of 4 repeat tests (intra-group) and 6 repeat tests (inter-group). NC1, 24 CFU/mL *E. coli* as negative control 1. NC2, 24 CFU/mL *S. Typhi* as negative control 2. The reference line was set as the highest value of the negative controls as the threshold. **e**, LOD analysis of *S. aureus* detection by qPCR. f, Interference resistance and specificity testing of TCC. Specificity and interference resistance were studied at 24 CFU/mL pathogen concentration. 1:1:1 indicates equal concentrations of 24 CFU/mL for three bacteria. SA, *S. aureus*. SP, *S. pyogenes*. LM, *L. monocytogenes*. EC, *E. coli*. ET, *S. Typhi*. Curve fitting in a and c used average of three technical replicates (n=3). All assays were performed in three independent biological triplicates (n=3). One-way ANOVA and Turkey's multiple comparisons test were used for statistical analysis. ns = not significant > 0.05. Asterisks indicate significant differences (*$p \leq 0.05$, **$p \leq 0.01$, ***$p \leq 0.001$, ****$p \leq 0.0001$). Error bars represent mean ± SD.

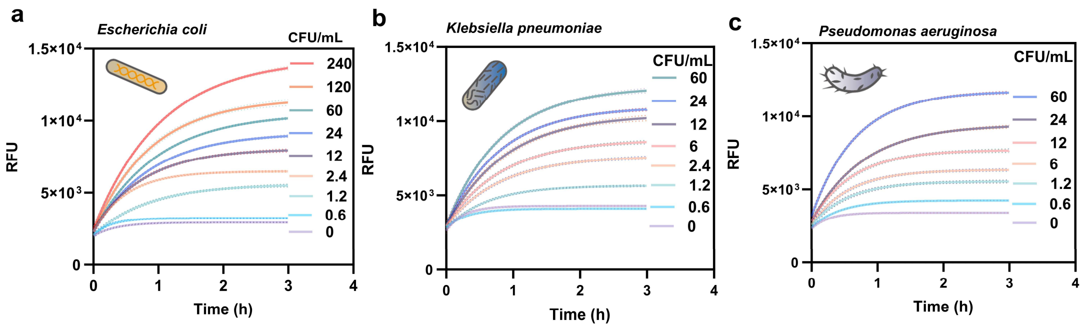

Supplementary Figure 15 | Detection of different pathogens using TCC.

a, Fluorescence kinetics of TCC detecting *E. coli*. **b**, Fluorescence kinetics of TCC detecting *K. pneumoniae*. **c**, Fluorescence kinetics of TCC detecting *P. aeruginosa*. Curve fitting in a, b and c was done by averaging values. All assays were performed in 3 independent biological triplicates (n=3), error bars represent mean ± SD.

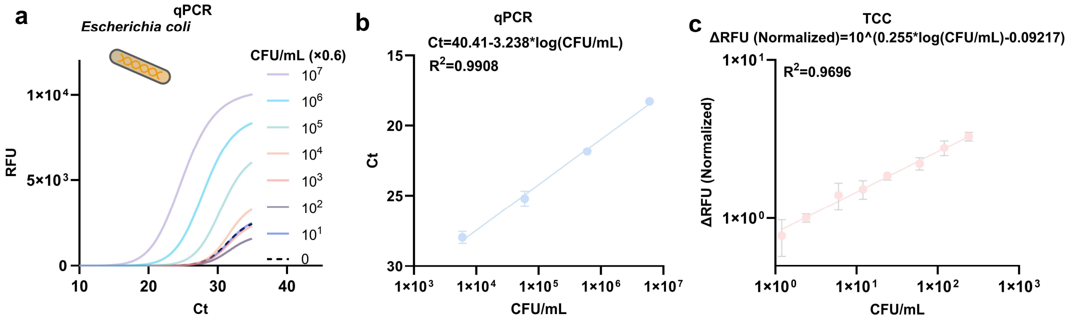

Supplementary Figure 16| qPCR and TCC quantification detection of simulated *E. coli* samples.

a, Ct-fluorescence kinetics of qPCR detecting *E. coli* at different concentrations. Curve fitting was done by averaging three technical repeats (n=3). **b**, Linear quantification of *E. coli* by qPCR. **c**, Linear quantification of *E. coli* by TCC. All assays were performed in 3 independent biological triplicates (n=3), error bars represent mean ± SD.

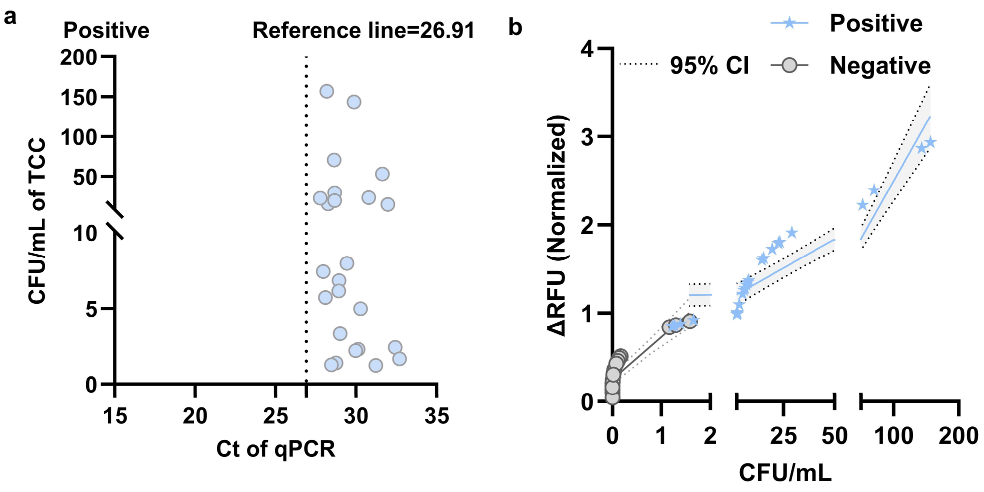

Supplementary Figure 17 | Detection of *E. coli* in serum by TCC and qPCR.

a, Quantitative results obtained from TCC assays compared to Ct values from qPCR. The reference line represents DEPC H_2O used as a sample input. **b**, Quantitative determination of *E. coli* in all serum samples (Patient IDs 1-50) by TCC assays. All assays were performed in 3 independent biological triplicates (n=3), Take the average value for plotting.

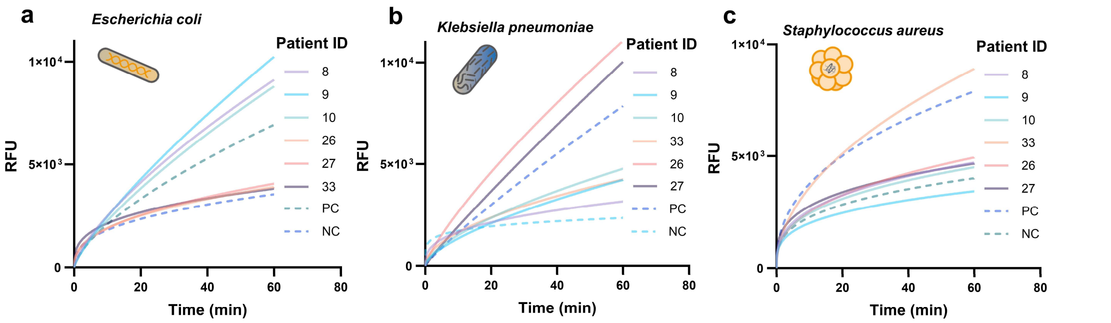

Supplementary Figure 18 | Fluorescence kinetics detection of different bacterial species from BSI patients.

a, Fluorescence kinetics of diagnosing *E. coli* (Patient IDs 8-10). **b**, Fluorescence kinetics of diagnosing *K. pneumoniae* (Patient IDs 26-27). **c**, Fluorescence kinetics of diagnosing *S. aureus* (Patient ID 33). PC, 2.4 CFU/mL positive control. NC, DEPC H_2O instead of target as negative control. All assays were performed in 3 independent biological triplicates (n=3). Linear fitting was done by averaging values.

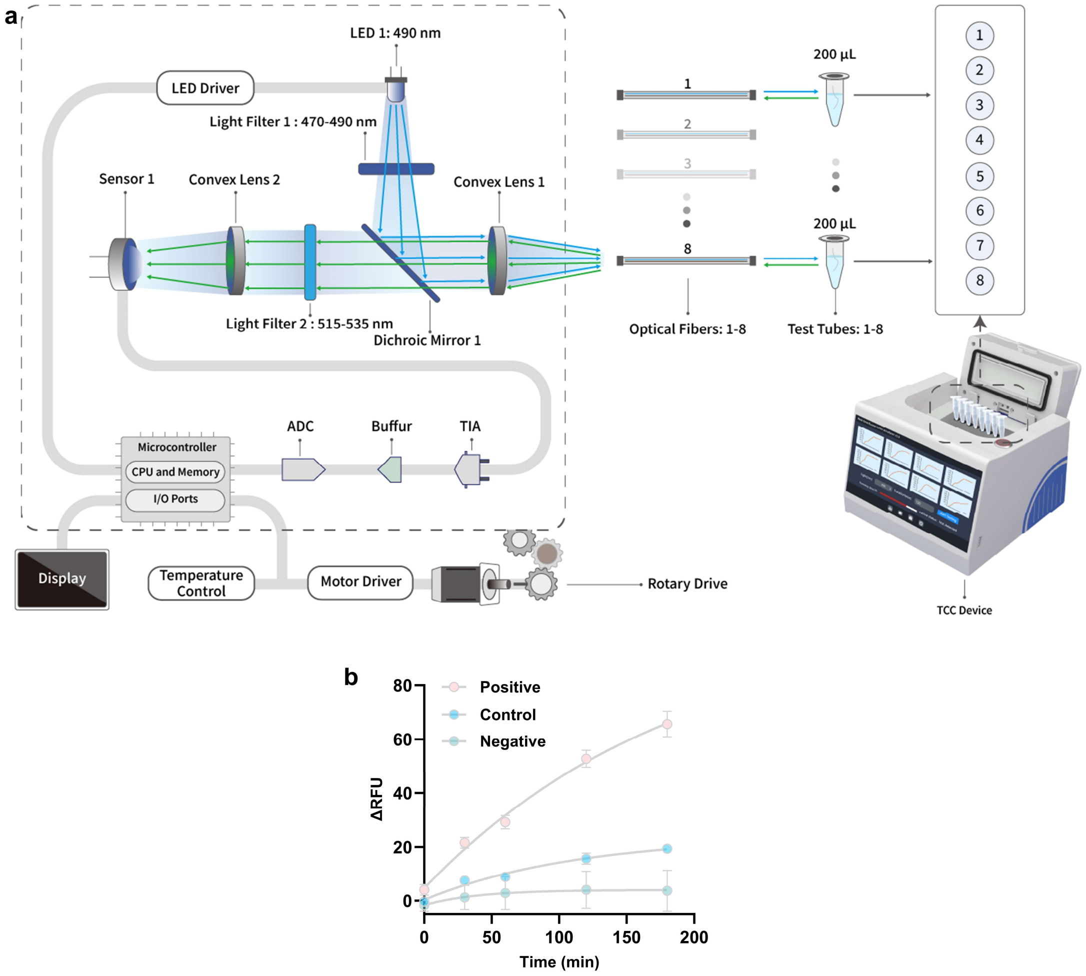

Supplementary Figure 19 | Principle and feasibility detection of the TCC Device.

a, Working principle of the TCC Device. Excitation light, the 490 nm LED light source 1 emits light through filter 1 to obtain high purity 490 nm excitation light, which is reflected by dichroic mirror 1 and focused by lens 1 onto the end of the optical fiber A for *trans*mission to directly irradiate the sample. Emission light, the 520 nm fluorescence emission from the sample, and background light are coupled into the optical path at optical fiber end B. Lens 1 *trans*forms the emission and background light into collimated beams to pass through dichroic mirror 1. Filter 2 selects the 520 nm fluorescence which is then focused by lens 1 onto the photosensitive area of the sensor that converts the optical signal into photocurrent proportional to the fluorescence intensity. The photocurrent is amplified by a TIA circuit with fluorescence voltage output passing through a buffer into the ADC, which digitizes the analog fluorescence data read directly by the microcontroller. The motor driver rotates the entire optoelectronic detection module (dashed box section) so that the focus of lens 1 coincides sequentially with the ends of optical fibers 1-8 to enable detection channel switching. **b**, Feasibility diagnosis of serum samples using the TCC Device. Positive, *E.coli* infected positive patient. Control, 2.4 CFU/mL cultured *E.coli*. Negative, *E.coli* infected negative patient. All tests were performed with 3 independent technical replicates (n=3), and error bars represent mean ± SD.

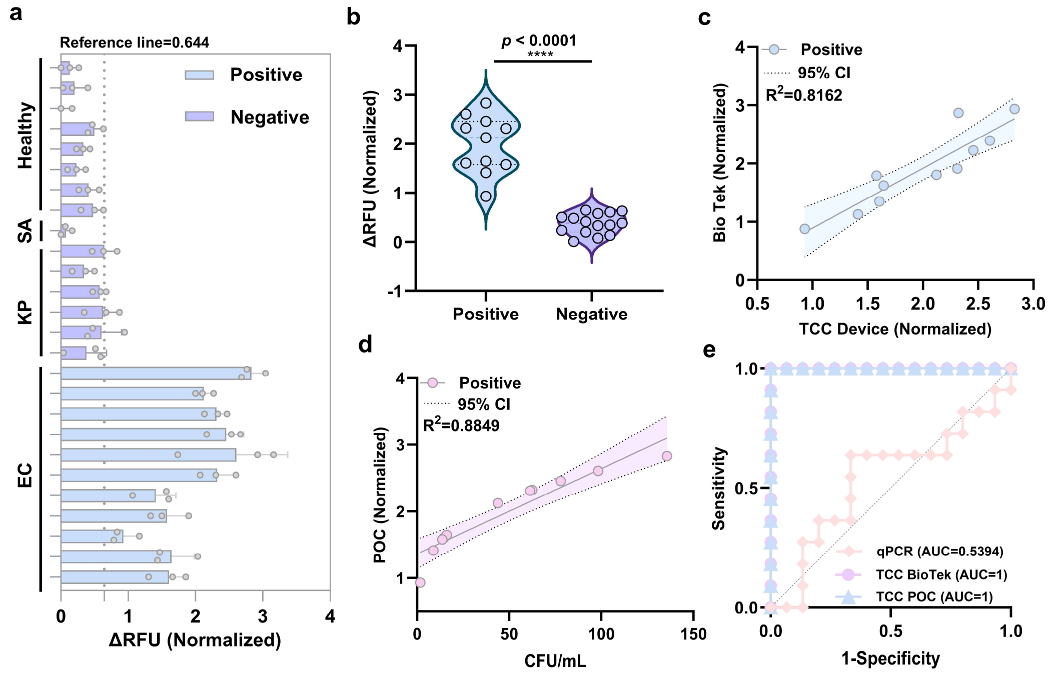

Supplementary Figure 20 | Point-of-care (POC) diagnosis of *E. coli* in serum by our own portable TCC Device.

a, Diagnosis of *E. coli* in serum by TCC Device. Reference line , the highest value of negative control (ΔRFU = 0.644) was used as the reference line. EC (Patient IDs 1-11), *E. coli*. KP (Patient IDs 25-30), *K. pneumoniae*. SA (Patient ID 33), *S. aureus*. **b**, Scatter plot of TCC Device diagnosis of *E. coli* in serum. statistical analysis of the diagnostic values for negative and positive patients was performed using an unpaired two-tailed t-test, where ns = not significant $p > 0.05$, and asterisks (*$p \leq 0.05$, **$p \leq 0.01$, ***$p \leq 0.001$, **$p \leq 0.0001$) indicate significant differences. **c**, Consistency evaluation between Bio Tek microplate reader and TCC Device for positive samples. 95% CI, 95% confidence interval. **d**, TCC Device quantification results of *E. coli* positive patients. Positive represents *E. coli* positive patient samples. **e**, ROC evaluation of the TCC device, Bio Tek microplate reader, and qPCR instrument for diagnosing *E. coli* in serum samples. All tests were performed with 3 independent technical replicates (n=3), and error bars represent mean ± SD.

References

1. Pausch, P., *et al.* CRISPR-CasΦ from huge phages is a hypercompact genome editor. *Science* **369**, 333-337 (2020).
2. Ramachandran, A. & Santiago, J.G. CRISPR Enzyme Kinetics for Molecular Diagnostics. *Analytical Chemistry* **93**, 7456-7464 (2021).
