# Supplementary material for: Ultrasensitive detection of clinical pathogens through a target-amplification-free collateral-cleavage-enhancing CRISPR-CasΦ tool
Source: Nat Commun. 2025 Apr 26;16:3929. doi: 10.1038/s41467-025-59219-x (PMC12032082; doi:10.1038/s41467-025-59219-x)
Supplement: Supplementary file 2 — Description of Additional Supplementary Files [file 41467_2025_59219_MOESM2_ESM.pdf]

Description of Additional Supplementary Files

File Name: Supplementary Data 1

Description: Nucleic acid sequences used in this study.

File Name: Supplementary Data 2

Description: Serum Sample Information.
